# Supplementary material for: Unveiling the Bioactive Potential of the Invasive Jellyfish Phyllorhiza punctata Through Integrative Transcriptomic and Proteomic Analyses
Source: Biomolecules. 2025 Aug 4;15(8):1121. doi: 10.3390/biom15081121 (PMC12383608; doi:10.3390/biom15081121)
Supplement: Supplementary file 1 [file biomolecules-15-01121-s001.zip › Suplementary Material/FileS3/PEPFOLD4/XP_065058059_13565/clusters.html]

 PEP-FOLD clusters


Download archive of all models  here   

|  |  |  |
| --- | --- | --- |
| # | file | sOPEP |
| # cluster 1 - Size: 95 |  |  |
|  | XP\_065058059\_13565-model1 | -36.8614 |
|  | XP\_065058059\_13565-model1.1 | -36.8242 |
|  | XP\_065058059\_13565-model1.2 | -36.8161 |
|  | XP\_065058059\_13565-model1.3 | -36.7601 |
|  | XP\_065058059\_13565-model1.4 | -36.7529 |
|  | XP\_065058059\_13565-model1.5 | -36.6991 |
|  | XP\_065058059\_13565-model1.6 | -36.661 |
|  | XP\_065058059\_13565-model1.7 | -36.6236 |
|  | XP\_065058059\_13565-model1.8 | -36.5645 |
|  | XP\_065058059\_13565-model1.9 | -36.5509 |
|  | XP\_065058059\_13565-model1.10 | -36.528 |
|  | XP\_065058059\_13565-model1.11 | -36.5258 |
|  | XP\_065058059\_13565-model1.12 | -36.5194 |
|  | XP\_065058059\_13565-model1.13 | -36.5172 |
|  | XP\_065058059\_13565-model1.14 | -36.5087 |
|  | XP\_065058059\_13565-model1.15 | -36.5014 |
|  | XP\_065058059\_13565-model1.16 | -36.494 |
|  | XP\_065058059\_13565-model1.17 | -36.4861 |
|  | XP\_065058059\_13565-model1.18 | -36.4675 |
|  | XP\_065058059\_13565-model1.19 | -36.4634 |
|  | XP\_065058059\_13565-model1.20 | -36.4559 |
|  | XP\_065058059\_13565-model1.21 | -36.4527 |
|  | XP\_065058059\_13565-model1.22 | -36.4509 |
|  | XP\_065058059\_13565-model1.23 | -36.4293 |
|  | XP\_065058059\_13565-model1.24 | -36.4198 |
|  | XP\_065058059\_13565-model1.25 | -36.4119 |
|  | XP\_065058059\_13565-model1.26 | -36.4099 |
|  | XP\_065058059\_13565-model1.27 | -36.4076 |
|  | XP\_065058059\_13565-model1.28 | -36.4009 |
|  | XP\_065058059\_13565-model1.29 | -36.3935 |
|  | XP\_065058059\_13565-model1.30 | -36.3914 |
|  | XP\_065058059\_13565-model1.31 | -36.389 |
|  | XP\_065058059\_13565-model1.32 | -36.3554 |
|  | XP\_065058059\_13565-model1.33 | -36.3474 |
|  | XP\_065058059\_13565-model1.34 | -36.3467 |
|  | XP\_065058059\_13565-model1.35 | -36.3446 |
|  | XP\_065058059\_13565-model1.36 | -36.3444 |
|  | XP\_065058059\_13565-model1.37 | -36.3409 |
|  | XP\_065058059\_13565-model1.38 | -36.3345 |
|  | XP\_065058059\_13565-model1.39 | -36.3308 |
|  | XP\_065058059\_13565-model1.40 | -36.3304 |
|  | XP\_065058059\_13565-model1.41 | -36.3247 |
|  | XP\_065058059\_13565-model1.42 | -36.3232 |
|  | XP\_065058059\_13565-model1.43 | -36.3072 |
|  | XP\_065058059\_13565-model1.44 | -36.3049 |
|  | XP\_065058059\_13565-model1.45 | -36.3048 |
|  | XP\_065058059\_13565-model1.46 | -36.2948 |
|  | XP\_065058059\_13565-model1.47 | -36.2693 |
|  | XP\_065058059\_13565-model1.48 | -36.2654 |
|  | XP\_065058059\_13565-model1.49 | -36.2605 |
|  | XP\_065058059\_13565-model1.50 | -36.2586 |
|  | XP\_065058059\_13565-model1.51 | -36.2575 |
|  | XP\_065058059\_13565-model1.52 | -36.2456 |
|  | XP\_065058059\_13565-model1.53 | -36.2374 |
|  | XP\_065058059\_13565-model1.54 | -36.2372 |
|  | XP\_065058059\_13565-model1.55 | -36.2186 |
|  | XP\_065058059\_13565-model1.56 | -36.2115 |
|  | XP\_065058059\_13565-model1.57 | -36.2062 |
|  | XP\_065058059\_13565-model1.58 | -36.1963 |
|  | XP\_065058059\_13565-model1.59 | -36.1911 |
|  | XP\_065058059\_13565-model1.60 | -36.1876 |
|  | XP\_065058059\_13565-model1.61 | -36.1869 |
|  | XP\_065058059\_13565-model1.62 | -36.1797 |
|  | XP\_065058059\_13565-model1.63 | -36.1706 |
|  | XP\_065058059\_13565-model1.64 | -36.1637 |
|  | XP\_065058059\_13565-model1.65 | -36.1634 |
|  | XP\_065058059\_13565-model1.66 | -36.1629 |
|  | XP\_065058059\_13565-model1.67 | -36.1627 |
|  | XP\_065058059\_13565-model1.68 | -36.1618 |
|  | XP\_065058059\_13565-model1.69 | -36.157 |
|  | XP\_065058059\_13565-model1.70 | -36.1431 |
|  | XP\_065058059\_13565-model1.71 | -36.1429 |
|  | XP\_065058059\_13565-model1.72 | -36.1206 |
|  | XP\_065058059\_13565-model1.73 | -36.1139 |
|  | XP\_065058059\_13565-model1.74 | -36.099 |
|  | XP\_065058059\_13565-model1.75 | -36.089 |
|  | XP\_065058059\_13565-model1.76 | -36.0601 |
|  | XP\_065058059\_13565-model1.77 | -36.0496 |
|  | XP\_065058059\_13565-model1.78 | -36.0367 |
|  | XP\_065058059\_13565-model1.79 | -36.0283 |
|  | XP\_065058059\_13565-model1.80 | -36.0283 |
|  | XP\_065058059\_13565-model1.81 | -36.0147 |
|  | XP\_065058059\_13565-model1.82 | -36.0023 |
|  | XP\_065058059\_13565-model1.83 | -35.9983 |
|  | XP\_065058059\_13565-model1.84 | -35.9647 |
|  | XP\_065058059\_13565-model1.85 | -35.9598 |
|  | XP\_065058059\_13565-model1.86 | -35.9413 |
|  | XP\_065058059\_13565-model1.87 | -35.9295 |
|  | XP\_065058059\_13565-model1.88 | -35.9233 |
|  | XP\_065058059\_13565-model1.89 | -35.9107 |
|  | XP\_065058059\_13565-model1.90 | -35.8836 |
|  | XP\_065058059\_13565-model1.91 | -35.8764 |
|  | XP\_065058059\_13565-model1.92 | -35.8713 |
|  | XP\_065058059\_13565-model1.93 | -35.6585 |
|  | XP\_065058059\_13565-model1.94 | -35.5803 |
| # cluster 2 - Size: 1 |  |  |
|  | XP\_065058059\_13565-model2 | -34.6221 |
| # cluster 3 - Size: 4 |  |  |
|  | XP\_065058059\_13565-model3 | -34.3836 |
|  | XP\_065058059\_13565-model3.1 | -34.2201 |
|  | XP\_065058059\_13565-model3.2 | -34.1395 |
|  | XP\_065058059\_13565-model3.3 | -34.0666 |
|  |  |  |
